# Antidepressant effects of activation of infralimbic cortex via upregulation of BDNF and β-catenin in an estradiol withdrawal model

**DOI:** 10.1007/s00213-024-06610-z

**Published:** 2024-05-14

**Authors:** Jiali Chen, Yiying Zhou, Miaojun Lai, Yanping Zhang, Yifang Hu, Dingding Zhuang, Wenhua Zhou, Yisheng Zhang

**Affiliations:** 1 Department of Obstetrics, The Affiliated Lihuili Hospital of Ningbo University, Ningbo, 315040 P. R. China; 2 Zhejiang Provincial Key Lab of Addiction Research, The Affiliated Kangning Hospital of Ningbo University, Ningbo, 315201 P. R. China; 3 Department of Psychiatry, Ningbo Kangning Hospital, Ningbo, 315201 P. R. China

**Keywords:** Postpartum depression, Infralimbic cortex, Glutamate, γ-aminobutyric acid receptor, Brain-derived neurotrophic factor

## Abstract

**Rationale:**

Clinical and preclinical studies have demonstrated that estradiol withdrawal after delivery is one of important factors involved in the pathogenesis of postpartum depression (PPD). The infralimbic cortex (IL) is related to anxiety and mood disorders. Whether IL neurons mediate PPD is still unclear.

**Objectives:**

This study was to observe the antidepressant effect and expression of BDNF and β-catenin in IL by allopregnanolone (ALLO) treatment or the selective activation or inhibition of IL neurons using a chemogenetic approach in a pseudopregnancy model of PPD.

**Methods:**

Administration of estradiol combined with progesterone and the abrupt withdrawal of estradiol simulated the pregnancy and early postpartum periods to induce depression in ovariectomized rats. The relative expression levels of β-catenin and BDNF were observed by western blotting.

**Results:**

Immobility time was significantly increased in the forced swim test and open-arm movement was reduced in the elevated plus maze test in the estradiol-withdrawn rats. After ALLO treatment, the immobility time were lower and open-arm traveling times higher than those of the estradiol-withdrawn rats. Meanwhile, the expression level of BDNF or β-catenin in the IL was reduced significantly in estradiol-withdrawn rats, which was prevented by treatment with ALLO. The hM3Dq chemogenetic activation of pyramidal neurons in the IL reversed the immobility and open-arm travel time trends in the estradiol-withdrawal rat model, but chemogenetic inhibition of IL neurons failed to affect this. Upregulated BDNF and β-catenin expression and increased c-Fos in the basolateral amygdala were found following IL neuron excitation in model rats.

**Conclusions:**

Our results demonstrated that pseudopregnancy and estradiol withdrawal produced depressive-like behavior and anxiety. ALLO treatment or specific excitement of IL pyramidal neurons relieved abnormal behaviors and upregulated BDNF and β-catenin expression in the IL in the PPD model, suggesting that hypofunction of IL neurons may be involved in the pathogenesis of PPD.

## Introduction

As a subtype of major depressive disorder, postpartum depression (PPD) occurs at any time during pregnancy or the early postpartum period (within 4 weeks of delivery), according to the definition in the Diagnostic and Statistical Manual of Mental Disorders, Fifth Edition. PPD is characterized by feelings of worthlessness, insomnia, irritability, and severe anxiety (O’Hara and McCabe 2013) and may adversely affect the child and other family members (Letourneau et al. 2012; Netsi et al. 2018; Soe et al. 2016). Various risk factors such as biological and psychosocial factors may contribute to onset of PPD. Preclinical evidence suggest fluctuations in neurosteroid hormone, stress and hypothalamic-pituitary-adrenal (HPA) axis, neuroinflammation, GABAergic signaling and changes of genetics and epigenetics are involved in the pathophysiology of PPD (Payne and Maguire 2019). PPD prevalence is approximately 12% and causes the severe consequences in women health and economics and social burden (Woody et al. 2017). Thus, there is an urgent need to elucidate the potential neurobiological mechanisms underlying PPD.

Experimental elevations of estradiol and progesterone concentrations, and then abrupt withdrawal of estradiol, induced depression in patients with a history of PPD (Bloch et al. 2000), and an estrogen supplement appeared to improve the depression in patients with PPD (Ahokas et al. 2001). Galea et al. first established an animal model of PPD based on the pseudopregnancy and estradiol and progesterone withdrawal (Galea et al. 2001). The depressive-like behavior, such as immobility in the forced swim test, could be prevented by estradiol administration (Galea et al. 2001; Stoffel and Craft 2004). Besides, accumulating evidence suggests that allopregnanolone (ALLO), a progesterone-derived neurosteroid plays a key role in PPD development. The variations of ALLO levels in the hippocampus mitigates proestrous changes in anxiety and depressive behavior (Frye and Walf 2002) and ALLO reduces the duration of immobility in forced swim test (Holubova et al. 2014; Khisti et al. 2000). ALLO regulates HPA axis function via GABAergic signaling to improve the stress on PPD (Maguire 2019). Now, brexanolone, chemically identical to endogenous ALLO, is the only antidepressant medication approved for PPD by the Food and Drug Administration (FDA) (Meltzer-Brody et al. 2018). In one study, ALLO exerts antidepressant effects in a PPD mouse model of maternal separation with early weaning (Garcia-Baos et al. 2022), but whether it is effective in the pseudopregnancy, and estradiol withdrawal model is unknown.

The medial prefrontal cortex (mPFC) plays a critical role in mediating many physiological functions and is closely related to fear conditioning, stress, and mood disorders (McEwen and Morrison 2013). Impairment of the mPFC often leads to changes in maternal care behavior (Afonso et al. 2007; Febo et al. 2010), and hypofunction of the mPFC has been found in women with PPD (Moses-Kolko et al. 2010). Chronic gestational stress induces depression-like behavior, reduces the density of the dendritic spine, and alters spine morphology on the pyramidal neurons in the mPFC (Leuner et al. 2014). The infralimbic cortex (IL) belongs to the ventral part of the mPFC, which is involved in the adaptation of maternal care behavior during the early postpartum period (Pose et al. 2019).The vesicular transporters of GABA and glutamate in IL reduces with lower ALLO levels in a PPD mouse model of maternal separation (Garcia-Baos et al. 2022). Moreover, estradiol modulates IL morphology and synapses in adult female rodents (Galvin and Ninan 2014; Velazquez-Zamora et al. 2012). Thus, we hypothesized that dynamic alterations of gestational hormones during the pregnancy and postpartum periods may account for PPD development via its effects on IL neurons.

Aberrations in the canonical Wingless/Integrated (Wnt) pathway has been related to mood disorders, including depression (Voleti and Duman 2012). β-catenin, as an important signaling molecular of Wnt pathway, is involved in cognitive function, neurogenesis, and dendritic remodeling (Yu and Malenka 2003). For example, upregulation of β-catenin levels has been shown to be an efficacy indicator for antidepressant effects (Ge et al. 2016). Moreover, a previous study showed that treatment with antidepressants resulted in the upregulation of brain-derived neurotrophic factor (BDNF) and β-catenin expression in the mPFC (Xu et al. 2017). Studies on postmortem samples of suicide victims with major depressive disorder have shown a reduction of β-catenin expression levels in the PFC (Karege et al. 2012). However, the role of BDNF and β-catenin expression in the IL in a PPD animal model remains unknown. In the present study, the effects of ALLO treatment on the depression- or anxiety-like behaviors and the BDNF and β-catenin expression in the IL were observed in the estradiol-withdrawal model of PPD. We then evaluated whether selective regulation of pyramidal neurons in the IL affects the PPD-like phenotype using a chemogenetic approach.

## Materials and methods

### Animals

**Fig. 1:**
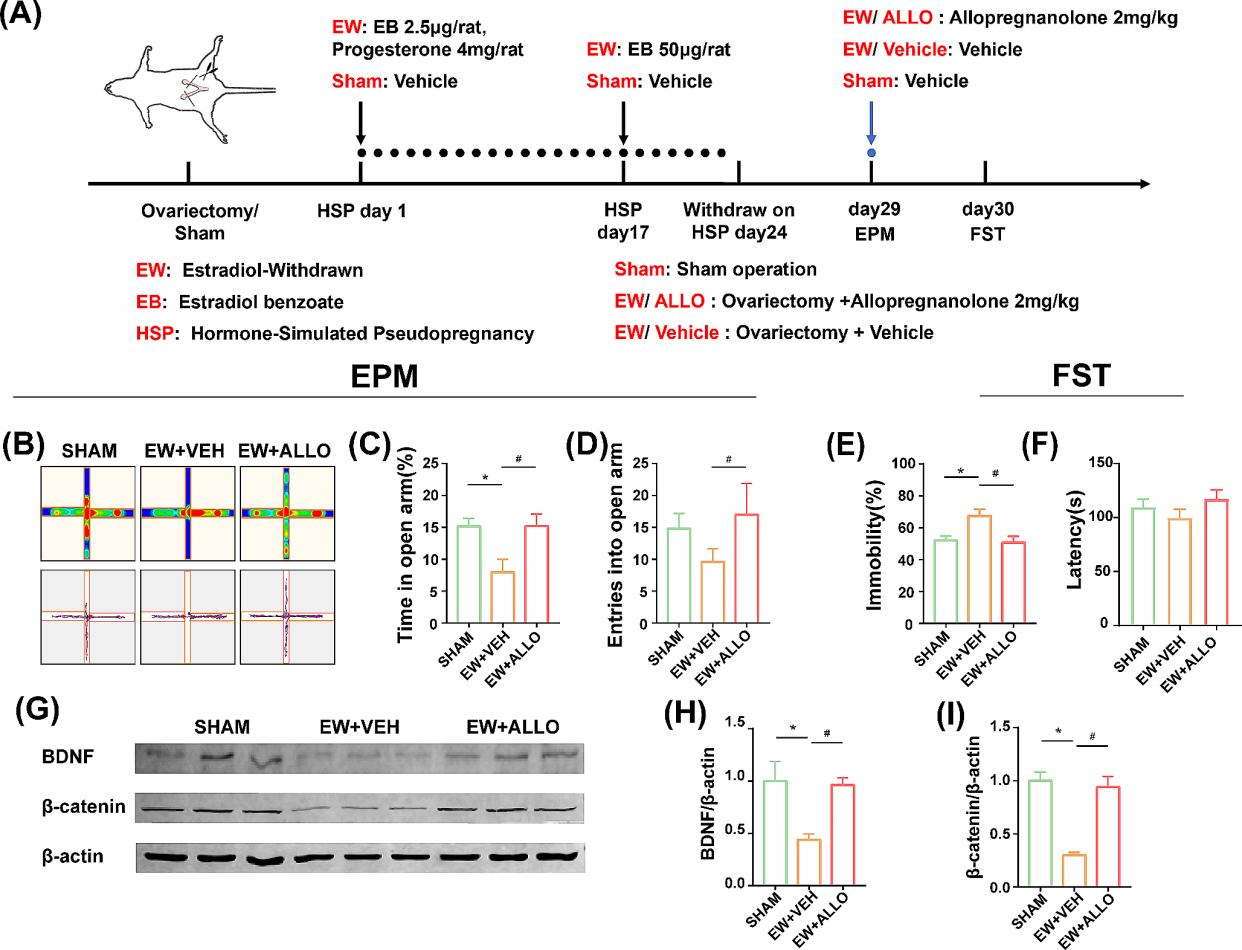
Effects of ALLO on depression- and anxiety-like behaviors and expression of BDNF and β-catenin in PPD model. (A) The experimental scheme of the estradiol withdrawal induced PPD rat model. From day 28 to 29 (withdrawal days 5 to 6), two behavioral tests are performed consecutively, and ALLO is administrated 2 h before each test. (B) Representative heat map and track across epochs in the EPM for rats (day 28). (C) The percentage of time spent in the open arm over a 5-min period (day 28). (D) The number of entrances into the open arm over a 5-min period (day 28).(E) The percentage of immobility time in the FST (day 29). (F) Representative western blotting of β-catenin and BDNF. (G) Quantification of relative expression of BDNF/β-actin. (H) Quantification of relative expression of β-catenin/ β-actin. Data are presented as mean ± SEM.* *P* < 0.05, compared with the Sham group, ^#^*P* < 0.05, compared with the EW + VEH group

**Fig. 2:**
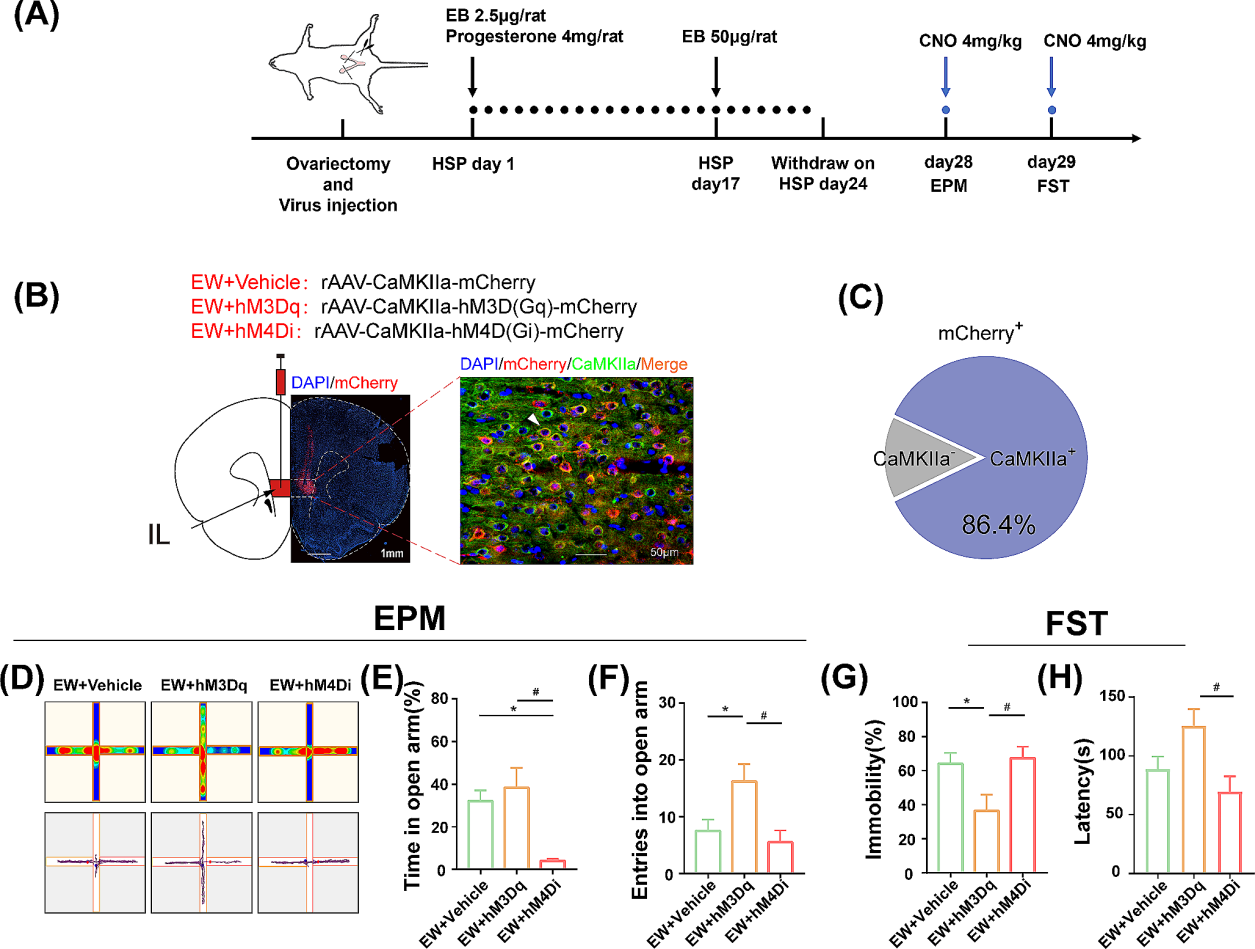
Effects of activating or inhibiting IL Pyramidal Neurons on depression- and anxiety-like behaviors in PPD Model. (A) The experimental timeline. (B) Schematic showing the virus injection into the IL. Left, mCherry expressed in the IL, Scale bar, 1 mm. Right, magnified image showing co-labeling of CaMKIIa^+^ neurons (green) and rAAV-mCherry (red) together with DAPI labeling for cell density (blue), and the co-localization of red and green staining (arrows), Scale bar, 50 μm. (C) 86.4% of the cells express both mCherry and CaMKIIa. (D) Representative heat map and track across epochs in the EPM for rats(day 28). (E) The percentage of time spent in the open arm(day 28). (F) The number of entrances into the open arm over a 5-min period. (G) The percentage of immobility time in the FST (day 29). Data are presented as mean ± SEM. * *P* < 0.05 compared with the vehicle group, ^#^*P* < 0.05, compared with the hM3Dq group. CNO, clozapine N-oxide

Female adult Sprague–Dawley rats (age: 8–10 weeks, *n* = 33, body weight: 250–280 g) were obtained from the Zhejiang Provincial Experimental Animal Center (Hangzhou, China) and housed in a pathogen-free environment, kept at a temperature of 22–24 °C and humidity of approximately 50–70%. All rats were housed in 45 × 25 × 15 cm polycarbonate cages with 2 or 3 rats per cage. All rats had free access to water and food and were under a 12 h light-dark cycle (lights on from 6 a.m. to 6 p.m.). The studies were carried out after 7 days, when all animals were acclimated to the new housing environment. The flowchart of the studies was presented in Figs. 1A and 2A. All animal studies and experimental protocols were approved by the Ethics Committee of Laboratory Animal Use and Care of Ningbo University.

### Drugs and viral vectors

Both estradiol benzoate and progesterone (Solarbio, Science & Technology Co., Ltd., Beijing, China) were dissolved in oil(Shanghai Macklin Biochemical Co., Ltd., Shanghai, China) as described previously (Galea et al. 2001). ALLO (Shanghai Macklin Biochemical Co., Ltd.) was dissolved in 35% 2-hydroxypropyl-β-cyclodextrin at the required concentration (2 mg/mL). Clozapine N-oxide (CNO) was purchased from MedChemExpress LLC (NJ, USA). Recombinant adeno-associated virus rAAV2/9- CaMKIIa-hM3D(Gq)-mCherry, rAAV2/9-CaMKIIa-hM4D(Gi)-mCherry, or a control vector (rAAV2/9-CaMKIIa-mCherry) were purchased from BrainVTA (Wuhan, China).

### Surgery

#### Ovariectomy

In experiment 1, the rats were assigned randomly to three groups (*n* = 5 per group): sham-operated (Sham), estradiol-withdrawn (EW + VEH), and ALLO-treated (EW + ALLO) groups. To remove endogenous sources of circulating gestational hormones, rats of EW + VEH and EW + ALLO groups were bilaterally ovariectomized (Fig. 1A). A single ventral midline incision was made to locate and extract both ovaries (Wu et al. 2020). Sham rats were anesthetized and subjected only to surgery without ovary removal.

#### Microinfusion

Three groups of rats (*n* = 6 per group, one rat dropped off platform in EPM in the hM3Dq group to incomplete the test) underwent simultaneous ovariectomy, were bilaterally injected rAAV vectors into the IL to express the control vector, hM3Dq or hM4Di in experiment 2, namely the vehicle, hM3Dq, or hM4Di groups. The vector was infused bilaterally into the IL by a microsyringe (Hamilton syringe, 5-µL) using following coordinates: an A/P (from bregma) + 3.0 mm; an M/L (from the midline), ± 0.5 mm; an D/V (from brain surface), -5.0 mm (Fig. 2B) as described previously(Chen et al. 2021b). A total of 0.5 µL virus solution was delivered to each location at speed of 0.1 µL/min via a micro-infusion pump (World Precision Instruments, Florida, USA).

All surgeries were performed using aseptic surgical techniques under anesthesia (Zoletil®50, also known as Telazol, is a ratio of 1:1 mixture of zolazepam and tiletamine, 50 mg/kg; Virbac, Carros, France). All animals were administered one subcutaneous injection of meloxicam (2 mg/kg, Meryer, Biochemical Technology Co., Ltd., Shanghai, China) for analgesic after surgery, then injection of penicillin (North China Pharmaceutical Company, Hebei, China) as an anti-inflammatory agent and recovered 7 days after the operation.

### Hormone-simulated pseudopregnancy (HSP)

After the rats recovered for 1 week after ovariectomy, the protocol of HSP was initiated, as shown in Fig. 1A. In this animal model, ovariectomized rats were administered a subcutaneous injection of estradiol benzoate (EB; 2.5 µg/rat) combined with progesterone (4 mg/rat) daily during the HSP at 9 o’clock in the morning for 16 consecutive days. A maximal dose of EB(50 µg/rat) dissolved in 0.1 ml sesame oil was administered alone for the next 7 days to mimic the 23-day gestational period in rats, as described previously(Galea et al. 2001). On day 24 (withdrawal day 1), the hormone injection was stopped, and hormone withdrawal was started. The Sham group was administered subcutaneously sesame oil alone at 0.l mL/day on the same schedule as the EB group.

### Drug treatment

In experiment 1, ALLO at a dose of 2 mg/kg was intraperitoneally administered to the EW + ALLO group two hours before the tests on day 28,29 (Fig. 1A), and the dosage of ALLO was chosen based on previous studies (Almeida et al. 2020; Rodriguez-Landa et al. 2007). Both the Sham and EW + VEH groups were administered an equal volume of 35% 2-hydroxypropyl-β-cyclodextrin (i.p. 1 mL/kg). In experiment 2, all rats received CNO (i.p. 4 mg/kg) on the same schedule (Fig. 2A).

### Behavioral tests

The EPM was performed on Day 28 and the FST was performed on Day 29. Both tests of Sham group were tested in the proestrus phase. The behavioral evaluations were conducted 2 h (Fig. 1A) after ALLO infusion in experiment 1 (Fig. 1A). In experiment 2, the behavioral evaluations were conducted 30 min after CNO infusion (Fig. 2A). All the behavioral scoring were performed blinded. After the last test was completed, the rats were anesthetized at once, then euthanized and collected brain tissue or perfused within one hour.

#### Elevated plus maze (EPM)

The EPM was elevated 50 cm from the ground and comprised four arms: two open arms and two closed arms (with walls). Each arm of open arms was 10 cm wide and 50 cm long while two closed arms had 30 cm walls. For testing, each rat was allowed free access to the center area of the maze, facing the same open arm, and allowed to explore the entire apparatus freely for 5 min (Ke et al. 2020; Suda et al. 2008). A video camera positioned over the top of the apparatus recorded the time spent in the open arm and the number of entrances into the open arm, and the percentage of time spent in the closed arm was calculated as an assessment of anxiety-like behavior. The maze was wiped off with alcohol before each test.

#### Forced swim test (FST)

A cylinder for FST (20 cm diameter and 50 cm height) was set at a water depth of 30 cm and temperature of 23–25 °C. The pretest wasn’t performed 24 h before of test. Each rat was placed in the cylinder and allowed to swim for 6 min, and the static time was recorded in the last 4 min. Each session was videotaped, and the immobility time was counted (Li et al. 2018). The time when the rat remained floating or motionless with only the movements necessary for maintaining balance in the water was defined as the immobility time, and the percentage of the immobility time refers to the percentage of the immobility time in last 4 min.

### Tissue collection

On day 29 (Figs. 1A and 2A), brain tissues (3 rats per each group) were isolated rapidly after anesthesia and stored at − 80 °C. As for immunolocalization of c-Fos or CaMKIIa, the rats were anesthetized and intracardially perfused with approximately 200 mL of saline, followed by 200 mL of 4% paraformaldehyde within one hour. Whole brains were immediately removed and postfixed in 4% paraformaldehyde overnight (4 °C) and then cryoprotected for 48 h in 30% sucrose in saline. Coronal slices (30 μm) of brain tissue were sectioned by using a frozen slicer (Leica, Wetzlar, Germany) and kept at − 20 °C until immunofluorescence labeling. Brain slices observed in IL with Bergmann from + 3.24 to + 2.76 and in basolateral amygdala (BLA) and central nucleus of the amygdala (CeA) with Bregman from − 2.4 to -2.64 were collected.

### Western blot

The rat was randomly chosen to performed Western Blot. The brain tissues were homogenized in radioimmunoprecipitation assay lysis buffer (500 µL/50 mg) containing phenylmethylsulfonyl fluoride (1 mM) and placed on ice for 30 min. Then, the supernatant was collected by centrifugation (12,000 rpm, 15 min, 4 °C). A bicinchoninic acid protein assay kit was used to analyze the total protein concentrations in the samples. The proteins of samples (40 µg) were electrophoresed by sodium dodecyl-sulfate polyacrylamide gel electrophoresis and transferred onto a polyvinylidene fluoride membrane (Bio-Rad, Hercules, CA, USA). The membranes were immersed in a washing buffer containing 5% fat-free milk for 2 h at room temperature on an agitator, followed by incubation in β-catenin (1:1000; 8480T, CST Medical Inc., Naples, FL, USA), BDNF (1:1000; ab6201, Abcam, Cambridge, UK), p-Glycogen synthase kinase 3 (GSK-3)β(Ser9) (1:1000; 5558 S, CST Medical Inc.), GSK-3β (1:1000; 12,456 S, CST Medical Inc.), p-cyclic adenosine monophosphate-response element binding protein (CREB)(Ser133) (1:1000; 9198 S, CST Medical Inc.), CREB (1:1000; ab32515, Abcam), P-p44/42 extracellular signal-regulated kinase (ERK) (T202/Y204, P-Erk1/2) (1:1000; 9101 S, CST Medical Inc.), p44/42 ERK (Erk1/2) (1:1000; 4695 S, CST Medical Inc.), p-AKT(Ser473) (1:2000; 4060 S, CST Medical Inc.), or AKT antibody (1:1000; 4685 S, CST Medical Inc.) overnight at 4 °C. ß-actin (1:5000; AC026, Abclonal, Woburn, MA, USA) was used as an internal control. Phosphorylated proteins such p-CREB, p-ERK, p-AKT or p-GSK-3β) were observed first, then the membranes were eluted with a stripping buffer, followed by incubation of the corresponding antibodies for CREB, ERK, AKT or GSK-3β as total protein expression. The membranes were washed the next day, then incubated with an Alexa Fluor 800 conjugated secondary antibody (1:5000) for 1 h at room temperature and visualized using a two-color infrared laser scanning imaging system (LI-COR Biotechnology, Lincoln, NE, USA). Image Studio Software (LI-COR Biotechnology) was used to quantify the gray value.

### Immunofluorescence

The slices in 10% bovine serum protein (containing 0.3% Triton X-100) at room temperature were incubated for 2 h. The primary antibodies, mouse anti-CaMKIIa (1:500; Abcam, ab22609) and rabbit anti-c-Fos (1:1000; Synaptic Systems GmbH, Goettingen, Germany) were added and incubated with the slices at 4 °C overnight. Next, the slices were washed thrice for 10 min in phosphate-buffered saline (PBST) and incubated with a secondary antibody (goat anti-rabbit conjugated to AlexaFluor488, 1:500; A11008, Invitrogen, Waltham, MA, USA or goat anti-mouse conjugated to CoraLite488, 1:500; SA00013, Proteintech, San Diego, CA, USA) for 2 h. Finally, the slices were washed with PBST and mounted with fluoroshield mounting medium containing 4’,6-diamidino-2-phenylindole (ab104139, Abcam). An Olympus BX53 microscope (Tokyo, Japan) was used for image acquisition, and the ImageJ software (National Institutes of Health, Bethesda, MA, USA) was used to calculate the number of cells labeled with red fluorescence (mCherry^+^) and co-labeled with yellow fluorescence (mCherry^+^ plus CaMKIIa^+^). Co-labeled rate (%) = number of cells with yellow fluorescence / total number of cells with red fluorescence.

### Statistical analysis

All statistical data were analyzed using GraphPad Prism 7.0 software (San Diego, CA, USA), shown as the mean ± standard error of the mean. The difference among group means was evaluated by using one-way analysis of variance (ANOVA) with Tukey’s multiple comparisons or using unpaired t-tests and were considered significant when *P* < 0.05.

## Results

### ALLO reversed the abnormal phenotype and loss of BDNF and β-catenin in the IL in PPD model

In experiment 1, the EPM were performed on day 28. One-way ANOVA revealed significant difference in the percentage of time spent in the open arm (Fig. 1C, F_(2,12)_ = 5.646, *P* = 0.0187) and the number of entries into the open arm (Fig. 1D, F_(2,12)_ = 6.371, *P* = 0.013) among three groups. The *post hoc* comparison showed a reduced percentage of time spent in the open arm (*P* = 0.033) or number of entries into the open arm (*P* = 0.074) in the EW + VEH group compared with sham group. However, ALLO-treated rats showed a higher percentage of time spent in the open arm (*P* = 0.0321) and number of entrances into the open arm (*P* = 0.0118) than those of the EW + VEH rats. The FST were performed on day 29, one-way ANOVA statistics showed a significant difference of the percentage of the immobility time among the three groups (Fig. 1E, F_(2,12)_ = 6.539, *P* = 0.0120). There is a significant increase of the percentage of the immobility time in EW + VEH group compared with the Sham group (*P* = 0.028), in contrast, the percentage of the immobility time was significantly decreased in ALLO treated group compared with the EW + VEH group (*P* = 0.0176).

To further observe the relationship between ALLO and IL function, the relative levels of BDNF and β-catenin in the ILs of each group was assessed (Fig. 1F). One-way ANOVA statistics revealed a significant difference in the levels of BDNF (Fig. 1G, F_(2,6)_ = 6.833, *P* = 0.0284) and β-catenin (Fig. 1H, F_(2,6)_ = 25.85, *P* = 0.0011) among the three groups. The multiple comparisons showed that relative levels of BDNF (*P* = 0.0375) and β-catenin (*P* = 0.0016) in the IL were reduced significantly in the EW + VEH group compared with sham group, while relative levels of BDNF (*P* = 0.0488) and β-catenin (*P* = 0.0025) in the EW + ALLO group were increased significantly compared with the EW + VEH group.

### Activation of IL pyramidal neurons reversed abnormal phenotypes in PPD model

To directly test whether the modulation of pyramidal neurons in the IL produces antidepressant and anxiolytic effects, we used a chemogenetic approach to specifically activate or inhibit pyramidal neurons (Fig. 2A). We observed strong selective mCherry expression in IL neurons (Fig. 2B and C). The distribution of mCherry + neurons resembled that of CaMKIIa-immunopositive neurons and demonstrated that the application of the rAAV virus produced an efficient and selective expression of hM3Dq/hM4Di in IL glutamatergic neurons.

To determine the influence of neuronal activation and silencing on behavioral responses, CNO was infused, and behavior was assessed 30 min after dosing. One-way ANOVA showed a remarkable difference in the percentage of time spent in the open arm (Fig. 2E, F_(2,14)_ = 10.48, *P* = 0.0016) and the number of entries into the open arm (Fig. 2F, F_(2,14)_ = 5.502, *P* = 0.0173) among the three groups. The multiple comparisons showed that the percentage of time spent in the open arm increased in the hM3Dq group compared with the hM4Di group(*P* = 0.0075), increased but not different with that of vehicle group(*P* > 0.05), while the percentage of time spent in the open arm decreased in the hM4Di group compared with the vehicle group (*P* = 0.0024).Moreover, the number of entries into the open arm increased in the hM3Dq group compared with the vehicle group (unpaired t-test, *P* = 0.036; one-way ANOVA, *P* = 0.0546) and with the hM4Di group (*P* = 0.0179). Moreover, one-way ANOVA showed a remarkable difference in percentage of immobility time among the three groups in the FST (Fig. 2G, F_(2,15)_ = 4.866, *P* = 0.0235), and multiple comparisons indicated that percentage of immobility time was reduced in the hM3Dq group compared with the vehicle group (unpaired t-test, *P* = 0.037;one-way ANOVA, *P* = 0.0559) and with the hM4Di group (*P* = 0.0314).

### Activation of IL pyramidal neurons reversed loss of BDNF and β-catenin in PPD model

**Fig. 3:**
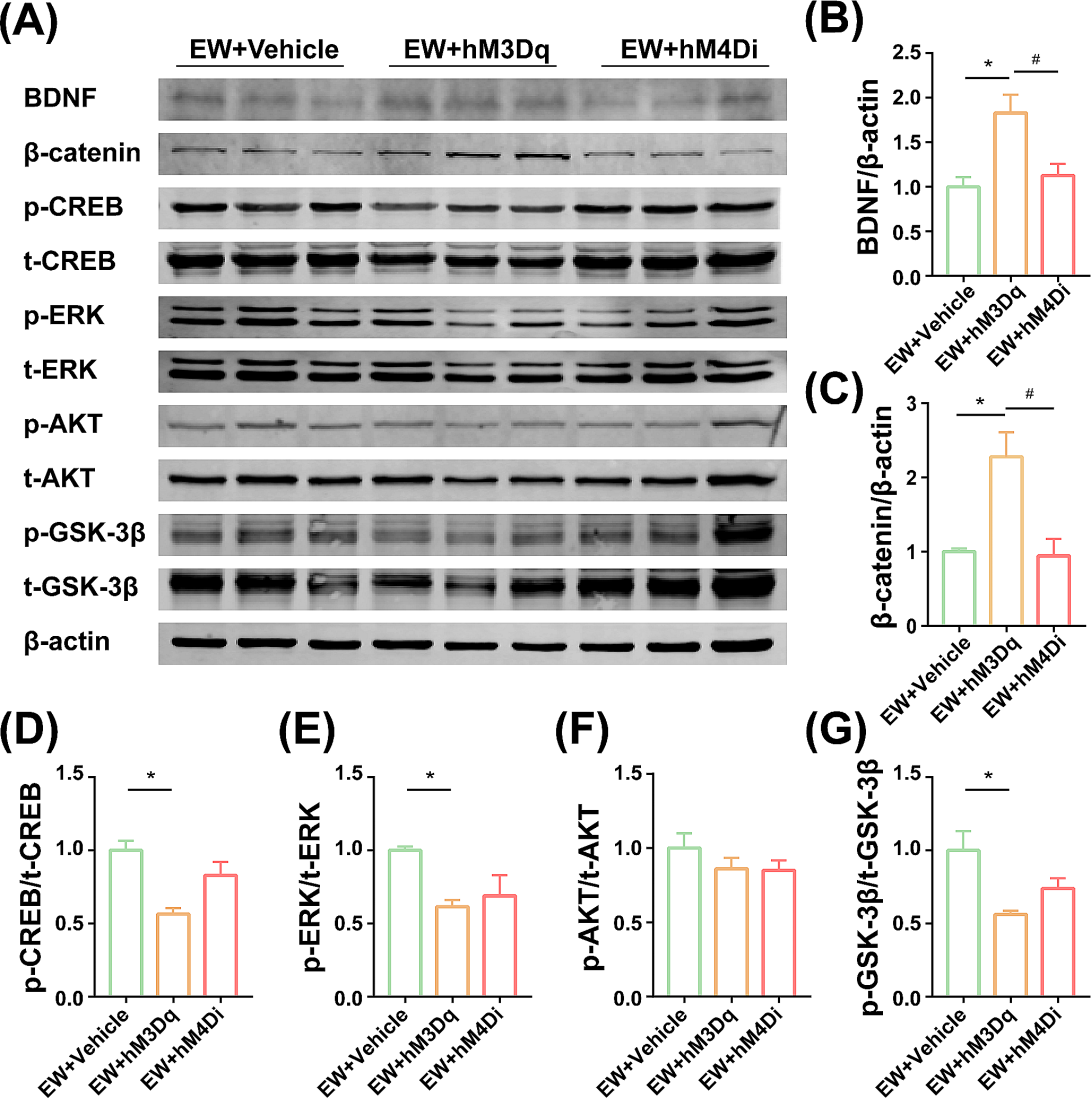
Effects of activating or inhibiting IL Pyramidal Neurons on IL-protein Expressions in PPD Model. (A) Representative western blotting of β-catenin, BDNF, p-CREB, t-CREB, p-ERK, t-ERK, p-AKT, t-AKT, p-GSK-3β, t-GSK-3β, and β-actin in the IL. (B) Representative western blots and levels of BDNF. (C) Representative western blots and levels of β-catenin. (D) Representative western blots and levels of p-CREB. (E) Representative western blots and levels of p-ERK. (F) Representative western blots and levels of p-AKT. (G) Representative western blots and levels of p-GSK-3β. Protein levels are normalized to vehicle values. Values represent the means ± SEM. * *P* < 0.05, compared with the vehicle group, ^#^*P* < 0.05, compared with the hM3Dq group

The relative expression in the IL was significantly different for BDNF (Fig. 3B, F_(2,6)_ = 8.496, *P* = 0.0178), β-catenin (Fig. 3C, F_(2,6)_ = 10.5, *P* = 0.0110), phosphorylation of CREB (Fig. 3D, F_(2,6)_ = 9.902, *P* = 0.0126), phosphorylation of extracellular signal-regulated kinase (ERK) (Fig. 3E, F_(2,6)_ = 5.473, *P* = 0.0444), and phosphorylation of GSK-3β (Fig. 3G, F_(2,8)_ = 6.285, *P* = 0.0337) among the three groups. However, there were no significant effects on the phosphorylation of AKT (Fig. 3F, F_(2,6)_ = 1.063, *P* = 0.4026). Both BDNF and β-catenin were expressed in higher levels in hM3Dq rats compared with the vehicle group (BDNF, *P* = 0.0203; β-catenin, *P* = 0.0191), with the hM4Di group (BDNF, *P* = 0.0405; β-catenin, *P* = 0.0158). Meanwhile, the activation of glutamatergic neurons in the IL decreased the phosphorylation of CREB (*P* = 0.0107), ERK (*P* = 0.0469), and GSK-3β (*P* = 0.0289) in the hM3Dq group compared with the vehicle rats, but not with the hM4Di group.

### Activation of IL neurons increased expression of c-fos and BDNF/β-catenin in the BLA

**Fig. 4:**
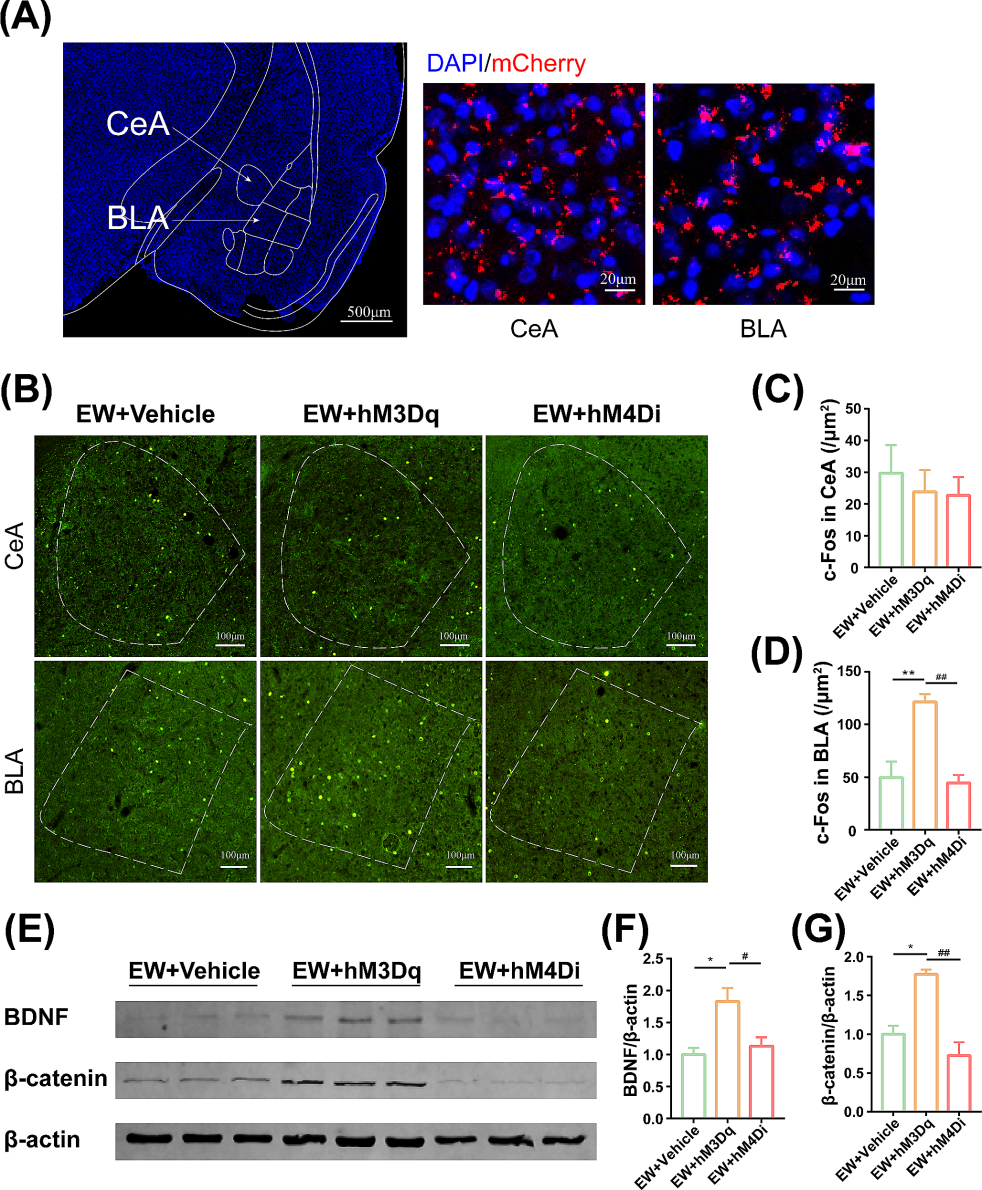
Expression of c-Fos and BDNF/ β-catenin in the BLA by exciting or inhibiting IL neurons in PPD Model. (A) Representative images showing mCherry expressed in the BLA and CeA. Scale bars, 500 μm (left), 20 μm (right). (B) Representative images for c-Fos immunofluorescence staining. Scale bars, 100 μm. (C) Number of c-Fos positive cells in the CeA. (D) Number of c-Fos positive cells in the BLA. (E) Representative western blotting of β-catenin, BDNF, and β-actin. (F) Representative western blots and levels of BDNF in the BLA. (G) Representative western blots and levels of β-catenin in the BLA. Protein levels are normalized to vehicle values. Values represent the means ± SEM. * *P* < 0.05, **P < 0.01 compared with the vehicle group, ^#^*P* < 0.05, ^##^*P* < 0.01 compared with the hM3Dq group

We first observed the red fluorescence of the virus in IL as axons labeling in the BLA and CeA, indicating that the BLA and CeA were subjected to glutamatergic projections from the IL (Fig. 4A). As a measure of neuronal activation, c-Fos immunoreactivity in the BLA and CeA was determined following chemical stimulation. The results showed that number of c-Fos positive neurons in the BLA was enhanced in the hM3Dq group compared with the other two groups (Fig. 4D, F_(2,6)_ = 16.13, *P* = 0.0039), but not in the CeA regions (Fig. 4C, F_(2,6)_ = 0.268, *P* = 0.7735). The expression of BDNF and β-catenin in the BLA in the hM3Dq group, was higher than that in the other two groups (Fig. 4F, F_(2,6)_ = 8.063, *P* = 0.0199; Fig. 4G, F_(2,6)_ = 19.68, *P* = 0.0023). In contrast, the expression of BDNF and β-catenin in the BLA in the hM4Di group was not different from the vehicle group. Collectively, the activation of pyramidal neurons in the IL excited the activity of the neurons and upregulated the expression of BDNF and β-catenin in the BLA. As shown in Fig. 5, we provided a diagram of the potential mechanism for ALLO in the Treatment of PPD.

## Discussion

In our rat model, higher estradiol and progesterone were administered to induce a hormone-simulated pseudopregnancy in ovariectomized rats. These hormones were then withdrawn only for one week. The main concern of this model is that an ovariectomy may affect these behaviors. Now, the time of ovariectomy related to develop the anxiety and depressive behavior is about 3 and 6 weeks post-ovariectomy respectively (Puga-Olguin et al. 2019; Rodriguez-Landa 2022). Thus, the abrupt withdrawal of estradiol and progesterone induce a PPD-like phenotype may not account for ovariectomy (Galea et al. 2001). These model responses are in line with a previous study in which abnormal behavior was improved by ALLO treatment(Almeida et al. 2020; Khisti et al. 2000; Rodriguez-Landa et al. 2007). ALLO is a positive allosteric modulator of γ-aminobutyric acid (GABA)_A_ receptors and is synthesized endogenously in the brain. Downregulation of GABA biosynthesis has been shown to contribute to mood disorder development, such as the onset of depression and anxiety, supporting the link between GABAergic signaling and PPD (Chen et al. 2021a). Animal models have shown that fluctuations of GABA_A_ receptor subunits expression and function occur during the pregnancy and postpartum periods (Licheri et al. 2015; Maguire and Mody 2008; Mostallino et al. 2009). Moreover, ALLO, as a treatment for PPD, has been shown to reverse abnormal behaviors in animal models based on maternal separation with early weaning (Garcia-Baos et al. 2022). Our findings confirmed the antidepressant effects of ALLO in an estrogen withdrawal rat model and validated the estradiol withdrawal model of PPD.

Accumulating evidence indicates that BDNF and β-catenin play a critical role in the pathophysiology of depression (Voleti and Duman 2012). The decline of BDNF in the mPFC has been found in both clinical and preclinical studies in mental disorders related to estradiol fluctuations, such as premenstrual dysphoric disorder (Oral et al. 2015), PPD (Lee et al. 2021), and perimenopausal depression (Harder et al. 2022). Moreover, estradiol supplementation increases BDNF and estrogen receptor β levels in the mPFC of ovariectomized rats (Wu et al. 2020). Through its action on the estrogen receptor β pathway, estradiol increases the growth and stability of new dendritic spines in the intact cortex (Wang et al. 2018). Either estradiol or BDNF induces the synaptic plasticity through fast membrane effects and produces slow transcriptional regulation through the CREB signaling pathway (Luine and Frankfurt 2013). β-catenin is a key molecule in the Wnt signaling pathway. A previous study has indicated that β-catenin levels in the postmortem prefrontal cortices decreased in patients with depression (Karege et al. 2012). In addition, some studies have pointed out that the interaction between estradiol and the Wnt signaling pathway mediates cognitive function (Fortress and Frick 2016). Furthermore, oligosaccharides extracted from *Morinda officinalis* exhibits an antidepressant effect via increased levels of BDNF and β-catenin in the mPFC (Xu et al. 2017). Our study found an abnormal expression of β-catenin and BDNF, both associated with depressive disorders, suggesting that the downregulation of β-catenin and BDNF in the IL region may be involved in the pathogenesis of PPD.

An intravenous formulation of ALLO (Brexanolone) has been approved specifically for the treatment of PPD by the United States FDA (Meltzer-Brody et al. 2018), and clinical trials on patients with PPD and interim results further support the significant reduction in depression symptoms (Walkery et al. 2021). Our results showed that ALLO had a rapid effect on depression/anxiety in an estradiol withdrawal model. ALLO inhibits depression and anxiety via GABAergic mechanisms (Chen et al. 2021a). GABA levels are negatively correlated with depression severity in subjects at risk for developing PPD (Payne and Maguire 2019). Normally, a rapid reduction in ALLO levels decreases the chloride influx and sensitivity of GABA_A_ receptors, hinders the inhibitory effect of GABAergic interneurons on the glutamatergic neurons, and decreases the excitability of pyramidal neurons in the IL (Gao et al. 2023). In contrast, ALLO increases the frequency of spontaneous glutamatergic postsynaptic excitatory currents through the potentiation of presynaptically located GABA_A_ receptors. ALLO exerts antidepressant effects by indirectly activating glutamatergic transmission; therefore, increasing excitatory glutamatergic neurons (Bali and Jaggi 2014).

The IL is an important region associated with cognition, with glutamatergic neurons accounting for the largest proportion (80%)(Chen et al. 2021b). Using a chemogenetic tool, the excitement of glutamatergic neurons in the IL displayed an antidepressant-like effect in the estradiol withdrawal model. Perimenopausal women are at increased risk for mood disorders, which may be related to reduced glutamate levels in the mPFC (Yap et al. 2021). Vulnerability to PPD is associated with unique fluctuations in mPFC glutamate levels, with glutamate levels being lower for high-risk women during pregnancy and early postpartum (Ghuman et al. 2022). In addition, decreased levels of vesicular glutamatergic type 1 transporter were found in the IL of PPD-like animals (Garcia-Baos et al. 2022). Consistent with these findings, our results support the idea of glutaminergic dysregulation and GABAergic deficits in PPD, as well as the potential of ALLO therapy because of its GABAergic effects (Morrow et al. 2022) on fear memory acquisition, consolidation, and retrieval (Narvaes et al. 2022). Chemogenetic activation of IL neurons decreased the phenotypes of PPD, suggesting that hypofunction of pyramidal neurons contribute to the pathogenesis of PPD. The interaction between GABA interneurons and pyramidal neurons maintains a balance for the excitation of neurons. The increased BDNF expression induced by ALLO supplementation or by chemogenetic activation suggests that IL neuronal deficits may be involved in PPD.

GSK-3β is a regulator of glucose metabolism, cell survival, and inflammation. Although GSK-3β is an active cytoplasmic kinase, its activity is decreased via phosphorylation of the Ser9 residue. Lithium, an inhibitor of GSK-3β, has been widely used to treat mood disorders (Vazquez et al. 2021), implicating the role of GSK-3β in mood disorders (Costemale-Lacoste et al. 2016). Studies in mice have shown that ketamine at antidepressant doses rapidly inhibits the activity of GSK-3β in the PFC (Duman et al. 2019). Dysregulation of Wnt/GSK-3β signaling is associated with a lifetime of major depression (Karege et al. 2012). Consistent with previous reports, decreased GSK-3β activity allowed the assemblage of β-catenin and β-catenin-dependent gene transcriptional events in our study. Exogenous ALLO supplementation improves the depression- and anxiety-like behaviors in rats exposed to chronic stress and its beneficial effects are secondary to increased BDNF levels in the brain (Evans et al. 2012). We found that abnormal behaviors in this model rats were reversed by either ALLO treatment or the chemogenetic excitation of IL neurons and were accompanied by the upregulation of β-catenin and BDNF in the IL. However, the chemogenetic activation of IL neurons also inhibited the expression of phosphorylated CREB and ERK in the PPD model. In addition, BDNF, an immediate upstream regulator of ERK and CREB, is downregulated in parallel with depression (Wang and Mao 2019). A reasonable explanation is that slight differences in the mechanism might underlie PPD and major depression or may be due to the adaptation of IL neurons for the chemogenetic excitability of pyramidal neurons.

Many studies have indicated that altered activity and deficits in well-known limbic circuits, such as the amygdala and PFC, are associated with emotionally relevant stimuli in patients with PPD (Hare and Duman 2020). The PFC is connected to other brain regions, specifically the amygdala. This reciprocal link helps regulate anxiety or fear in both regions (Pillerova et al. 2022). The BLA is also strongly associated with emotional and cognitive regulation. It has been found that effective connections from the PFC to the amygdaloid nucleus are reduced in patients with PPD (Garcia-Baos et al. 2022). Previous studies have shown that IL-to-BLA projections are involved in fear extinction (Park and Chung 2020) and social behavior (Huang et al. 2020). Recently, evidence has indicated a key role of GABA interneurons in oscillations generated in the BLA and that ALLO increases the high-theta oscillations (6–12 Hz) via the delta-containing GABA_A_ receptors in the BLA. Moreover, theta oscillations in the BLA are correlated with protection against disruption of the network and behavioral states induced by chronic stress (Antonoudiou et al. 2022). In the present study, the results showing the activation of BLA neurons with enhanced c-Fos positivity and the upregulation of BDNF/β-catenin in the BLA after IL excitation demonstrated that the IL projects to the BLA, mediating the well-established anxiolytic and antidepressant effects.

Studies on the neurobiological mechanisms underlying major depression have focused on the balance of excitatory pyramidal neurons and inhibitory GABA interneurons; both could result in the signal integrity in cortical function (Duman et al. 2019). The disinhibition hypothesis states that this increasing release of glutamate is due to the ketamine antagonism of N-methyl-D-aspartate (NMDA) receptors on tonically active GABAergic interneurons. Blockage of these NMDA receptors subsequently eliminates the inhibition (i.e., disinhibition) of glutamatergic neurons, thereby allowing excitement of pyramidal neurons within the mPFC (Sial et al. 2020). Lower glutamate concentration in the mPFC is related to an increased risk of early onset PPD in biologically vulnerable women (Ghuman et al. 2022). Based on the GABAergic deficits implicated in major depressive disorder, these findings provide insight into the mechanisms of ALLO targeting on GABAergic transmission and highlight the potential role of GABAergic plasticity in the pathogenesis of PPD (Gunduz-Bruce et al. 2022). The results of our study suggest that deficits of functional GABA_A_ receptors may underlie the biological mechanisms that contribute to the manifestation of PPD induced by estradiol withdrawal.

In conclusion, our study demonstrated that estradiol withdrawal elicited a depression- and anxiety-like state in rats and downregulated the BDNF/β-catenin expression in the IL, which could be reversed with ALLO supplementation. Neuronal activation in the IL by using a chemogenetic tool reversed the depression- and anxiety-like phenotypes induced by estradiol withdrawal and induced accordingly the upregulation of BDNF/β-catenin in the IL and BLA. These results suggest that ALLO may regulate BDNF/β-catenin expression in the IL via GABAergic signaling to play anxiolytic- and antidepressant-like effects in an estradiol withdrawal model, and hypofunction of IL neurons may be involved in the pathogenesis of PPD.
